# Circular RNA expression profiles in extracellular vesicles from the plasma of patients with pancreatic ductal adenocarcinoma

**DOI:** 10.1002/2211-5463.12741

**Published:** 2019-11-15

**Authors:** Qingqing Li, Shasha Geng, Huixiao Yuan, Yang Li, Shuxian Zhang, Lin Pu, Jianli Ge, Xianping Niu, Yandong Li, Hua Jiang

**Affiliations:** ^1^ Shanghai East Hospital Nanjing Medical University Shanghai China; ^2^ Department of Geriatrics Shanghai East Hospital Tongji University School of Medicine Shanghai China; ^3^ Department of Gastroenterology The First People’s Hospital of Lianyungang China; ^4^ Research Center for Translational Medicine Shanghai East Hospital Tongji University School of Medicine Shanghai China

**Keywords:** circular RNA, extracellular vesicles, microRNA, pancreatic ductal adenocarcinoma

## Abstract

Tumor‐derived extracellular vesicles (EVs) and their contents are involved in the development of human malignancies. Circular RNAs (circRNAs), enriched in EVs, can regulate diverse cellular processes by acting as microRNA (miRNA) sponges or through other mechanisms. In the present study, we explored the potential roles of circRNAs in EVs in the development of pancreatic ductal adenocarcinoma (PDAC). First, plasma was obtained from patients with PDAC (*n* = 8) and healthy volunteers (*n* = 8), and EVs were isolated by the ultracentrifugation method. Nanoparticle tracking analysis and transmission electron microscopy confirmed the size and form of the isolated EVs. The circRNA expression profiles of EVs were investigated by high‐throughput whole transcriptome sequencing. We then further validated the accuracy of the circRNA sequencing data by quantitative real‐time PCR analysis using plasma samples and PC cell lines, and subsequently performed bioinformatics analysis to reveal the potential functional roles of the differentially expressed circRNAs and to construct a circRNA‐miRNA interaction network to predict the target miRNAs of these circRNAs. Our work provides novel targets for further studies concerning the pathogenesis of PDAC.

## Abstract 2

Extracellular vesicles are frequently linked to a variety of human cancers. In this study, sequencing of circular RNA (circRNA) in extracellular vesicles reveals abundant circRNA in human plasma as potential biomarkers for pancreatic cancer diagnosis. Moreover, the accuracy of the circRNA sequencing data was further validated by quantitative real‐time PCR analysis using plasma samples and PC cell lines.

## Graphical abstract

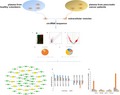

**Abbreviations**
BP: biological process
CC: cellular component
circRNA: circular RNA
circRNA‐seq: circular RNA sequencing
EV: extracellular vesicle
GO: Gene Ontology
KEGG: Kyoto Encyclopedia of Genes and Genomes
MF: molecular function
miRNA: microRNA
NTA: nanoparticle tracking analysis
PDAC: pancreatic ductal adenocarcinoma
RNA‐seq: RNA sequencing
TEM: transmission electron microscope

Pancreatic ductal adenocarcinoma (PDAC) is the fourth leading cause of cancer‐related death in the United States, with an estimated 45 750 deaths of this disease in 2019 1. Late diagnostic capabilities, early metastasis and a fast developing chemoresistance lead to a poor prognosis of patients with PDAC, with a 5‐year survival rate of less than 5% 2. Therefore, a better understanding of the molecular mechanisms behind the carcinogenesis and development of PDAC is essential for developing effective therapies against PDAC.

Extracellular vesicles (EVs) are 30‐ to 120‐nm phospholipid bilayer‐enclosed membrane nanoparticles that are released from various types of cell and are found in abundance in body fluids including blood, saliva, urine and breast milk 3, 4, 5. The components of EVs vary according to different cell types and mechanism of biogenesis, including lipid, proteins, mRNAs, DNA fragments and noncoding RNAs 6, 7, 8, 9. Accumulating evidence has indicated that EVs are essential for intercellular communication and participate in many physiological and pathological processes 10, 11, 12, and importantly, EVs derived from tumor cells have been found to play key roles in the development of human malignancies by transferring their components into target cells to regulate tumor initiation, tumor cell growth, metastasis, immune response, systemic cell‐to‐cell relevance and so on 13, 14. For instance, Melo *et al*. 15 found GPC1^+^ circulating exosomes were highly expressed in the serum of patients with pancreatic cancer and correlated with tumor burden, exosome‐mediated transfer of miR‐10b was reported to promote cell invasion of breast cancer 16. Among the components of exosomes, circular RNAs (circRNAs) are a novel type of noncoding RNA, which is becoming a hot spot in cancer research 17, 18, 19. Unlike linear RNAs terminated with 5′ caps and 3′ tails, circRNAs form covalently closed continuous loop structures 20. They could act as microRNA (miRNA) sponges, regulators of splicing and transcription and modifiers of parental gene expression 20, 21, 22. In recent years, scientists have proved that circRNAs participate in some pathological process, such as prion infection 23, neurological disorders 24 and atherosclerotic vascular disease 25, and especially in various human malignancies 26, 27, 28. In 2015, Li *et al.* 29 first identified more than 1000 circRNAs in human serum exosomes and revealed that circRNAs were enriched in exosomes of liver cancer cells compared with the producer cells by RNA sequencing (RNA‐seq) analyses. A previously identified circRNA, ciRS‐7, could promote epidermal growth factor receptor expression by acting as a miRNA sponge of miR‐7, and epidermal growth factor receptor overexpression was related to tumor progression and resistance to chemotherapy 21, 30. However, due to the numerous types of pancreatic cancer–derived EV and diverse contents, little is known about EVs circRNAs in PDAC, and using them as potential biomarkers of PDAC is still at an exploratory stage.

In this study, EVs from plasma of patients with PDAC and healthy volunteers were isolated and characterized first, after which we evaluated the EVs circRNA expression profiles in PDAC (*n* = 8) and normal plasma samples (*n* = 8) by high‐throughput whole transcriptome sequencing. Furthermore, bioinformatic analysis was used to predict the function of differentially expressed circRNAs, and 13 differentially expressed circRNAs were selected to validate the accuracy of the RNA‐seq data by quantitative real‐time PCR analysis. In addition, a circRNA–miRNA interaction network was constructed. Our work could provide a basis for further studies concerning the functions of EVs circRNAs in PDAC.

## Materials and methods

### Sample collection

Blood samples (8 mL) from eight patients with PDAC and eight healthy volunteers were collected in Shanghai East Hospital from May 2017 to March 2018. All of the patients with PDAC were diagnosed according to the seventh edition of the *American Joint Committee on Cancer* staging manual and confirmed by pathologists after surgery, and they accepted neither radiotherapy nor chemotherapy before surgery. Fasting venous blood was drawn and centrifuged; the separated plasma was stored at −80 °C before the experiments. This study was approved by the Ethics Committee of Biomedicine Research at Shanghai East Hospital, Tongji University, and written informed consent was obtained from each participant in this study. The use of patient samples conformed to the guidelines set by the Declaration of Helsinki.

### Extraction and characterization of the PDAC‐derived EVs

The plasma samples were centrifuged at 1500 ***g*** for 5 min and then 10 000 ***g*** for 30 min at 4 °C, after which the debris was removed. Supernatant was filtered through a 0.22‐µm filter to remove large particles and then centrifuged at 150 000 ***g*** for 16 h to obtain the EVs. The pellet containing EVs was diluted with PBS and centrifuged at 150 000 ***g*** for 2 h. EVs were purified by density gradient ultracentrifugation, then stored at −80 °C before quantitation. All ultracentrifugation steps were performed in an Optima L‐100XP ultracentrifuge (Beckman Coulter, Brea, CA, USA). The EV samples were diluted with PBS (1 : 100) before diameter and concentration quantitation, and a NanoSight NS300 instrument (Malvern Instruments, Malvern, UK) was used for nanoparticle tracking analysis (NTA). Each sample was read in triplicate. The size and number of the vesicles were recorded by the NTA software (NanoSight Limited, Malvern Instruments, Amesbury, UK).

### Transmission electron microscopy

EVs were visualized using a transmission electron microscope (TEM) (JEM‐2100F; JEOL, Tokyo, Japan). In brief, samples for TEM were diluted to 1 mg·mL^−1^ in PBS and absorbed onto carbon/formvar TEM grids (400 mesh). After drying for 10 min at room temperature, the grids were stained with phosphotungstic acid solution (2%, pH 7.0) for 15 min. After further drying overnight at room temperature, the stained grids were observed and photographed using a TEM.

### RNA library construction and circRNA sequencing

For total RNA extraction from EVs, TRIzol reagent (Invitrogen, Carlsbad, CA, USA) was used according to the manufacturer’s instruction. The concentration and purity of each RNA sample were measured by NanoDrop ND‐1000 (Thermo Fisher Scientific, Waltham, MA, USA). Standard denaturing agarose gel electrophoresis was used to examine RNA integrity. RNA library construction and circRNA sequencing (circRNA‐seq) were performed by CloudSeq Biotech Inc. (Shanghai, China). To enrich circRNAs and deplete rRNAs, we treated the total RNA with RNase R (Epicentre, Madison, WI, USA) and the Ribo‐Zero Magnetic Gold Kit (Epicentre). RNA libraries were constructed using the rRNA‐depleted RNAs with TruSeq Stranded Total RNA Library Prep Kit (Illumina, San Diego, CA, USA) according to the manufacturer’s protocols. The BioAnalyzer 2100 system (Agilent Technologies, Santa Clara, CA, USA) was used to confirm the quality and quantity of the libraries. Libraries were denatured into single‐stranded DNA molecules, captured on Illumina Flow Cells (Illumina) and then amplified *in situ* as clusters, and finally sequenced for 150 cycles using the HiSeq 4000 Sequencing system (Illumina).

### Bioinformatics analysis

Gene Ontology (GO) (http://www.geneontology.org) and Kyoto Encyclopedia of Genes and Genomes (KEGG) pathway analyses (http://www.genome.jp/kegg) were performed for the differentially expressed circRNA‐associated genes. The coordinates of the mRNA (which is defined as the circRNA‐associated gene) were predicted through the RefSeq database based on back‐splicing site coordinates of the circRNA. The circRNA‐miRNA network was generated by cytoscape software (http://www.cytoscape.org/download.html).

### Quantitative real‐time PCR analysis

Quantitative real‐time PCR analysis was performed to verify the accuracy of the circRNA‐seq data. The target RNA and internal parameters of each sample were respectively subjected to quantitative real‐time PCRs on an Applied Biosystems 7500 Fast qRT‐PCR System (software version 2.0.5; Roche, Basel, Switzerland). Total RNA was reverse transcribed to synthesize cDNA using a Prime Script RT Reagent Kit (Perfect Real Time; TaKaRa, Osaka, Japan). The data were analyzed by the $2^{-\Delta \Delta C_{T}}$ method. An outward‐facing primer was typically designed for an exon sequence near the back‐splice site (within 150 nucleotides up or down) of the corresponding circRNA. Primers of the selected circRNAs and internal parameters of GAPDH were shown in Table 1. Thirteen differentially expressed circRNAs were used in experimental validation including eight up‐regulated circRNAs and five down‐regulated circRNAs. Moreover, expression of four up‐regulated circRNAs was detected in pancreatic cancer cells.

**Table 1:** Primers used for quantitative real‐time PCR analysis of circRNA levels. F, forward; PS, product size; R, reverse.

Target ID	Primer sequence (5′–3′)	PS (bp)
hsa_circ_0002130	F: CCACGTGGGAGATTCTGG	121
	R: ACGTTCCACAGCCAGCTC	
hsa_circ_0000896	F: TCCTGGAGATGGGGTTCA	233
	R: GCCAAGAGCCACTGGAGA	
hsa_circ_0101692	F: TACTCGGCCAGCGGTTTA	61
	R: TCCATTTCCCGGATAGCA	
hsa_circ_0005882	F: GCCCACCCAATGCTAATG	214
	R: TGACCACTTGACCCAGGAA	
hsa_circ_0001250	F: GACAGCGAGGAGCTGAGG	167
	R: CCTAACATGACGCTCGGG	
hsa_circ_0037096	F: GGCAAAGAGGTTGCTGGA	263
	R: TGTGAGATGGAGGTGCGA	
hsa_circ_0000128	F: ATAACAGTTGCTGCCGCC	163
	R: TTTCTTTCCTTTCCCGGC	
hsa_circ_0000606	F: ATTGCATTCCAACAGCTCA	60
	R: CCGGCCATCAACAATTTC	
hsa_circ_0103896	F: CCTGAACAGCCAGTGGAGA	174
	R: TCCTGGAGGAGGGGTTTC	
hsa_circ_0006662	F: TGGAAAAGAGCCGAGTGG	190
	R: CTGCAACCTTTCCTCCAGA	
hsa_circ_0092763	F: GCAGTATGCTGTGGAGGGA	215
	R: GGATAGCCTTCAATGAGCCA	
hsa_circ_0035432	F: GGCCCTGAGGGAGAGTGT	167
	R: AGGAGCCTGCCTTGGAGT	
hsa_circ_0094190	F: GGGCTGTCATCTGGTTGG	197
	R: CGATGCACTGCCACCTTA	
GAPDH	F: GGCCTCCAAGGAGTAAGACC	122
	R: AGGGGAGATTCAGTGTGGTG	

### Cell culture

Human pancreatic cancer cells (BxPC‐3, COLO‐357, PANC‐1 and SW1990) were purchased from the American Type Culture Collection (ATCC, Manassas, VA, USA). BxPC‐3 cells were cultured in RPMI 1640 medium (Corning, NY, USA) supplemented with 10% fetal bovine serum (Corning) and 100 U·mL^−1^ penicillin and streptomycin (Gibco, Grand Island, NY, USA), Colo‐357 cells were cultured in RPMI 1640 medium (Corning) supplemented with 20% fetal bovine serum (Corning) and 100 U·mL^−1^ penicillin and streptomycin (Gibco), and PANC‐1 and SW1990 cells were cultured in DMEM medium (Corning) supplemented with 10% fetal bovine serum (Corning) and 100 U·mL^−1^ penicillin and streptomycin (Gibco). All cell lines were incubated at 37 °C with 5% CO_2_. Medium was replaced every other day. At 80% confluence, adhered cells were digested with 0.25% trypsin.

### Statistical analysis

For statistical analysis, each experiment was repeated at least three times independently. Data were presented as the mean ± standard deviation. A two‐tailed Student’s *t*‐test was used to assess the difference between two groups. A *P*‐value <0.05 was considered statistically significant. All analyses were carried out using spss 19.0 software (SPSS, San Rafael, CA, USA).

## Results

### Detection and characterization of circulating EVs

**Figure 1:**
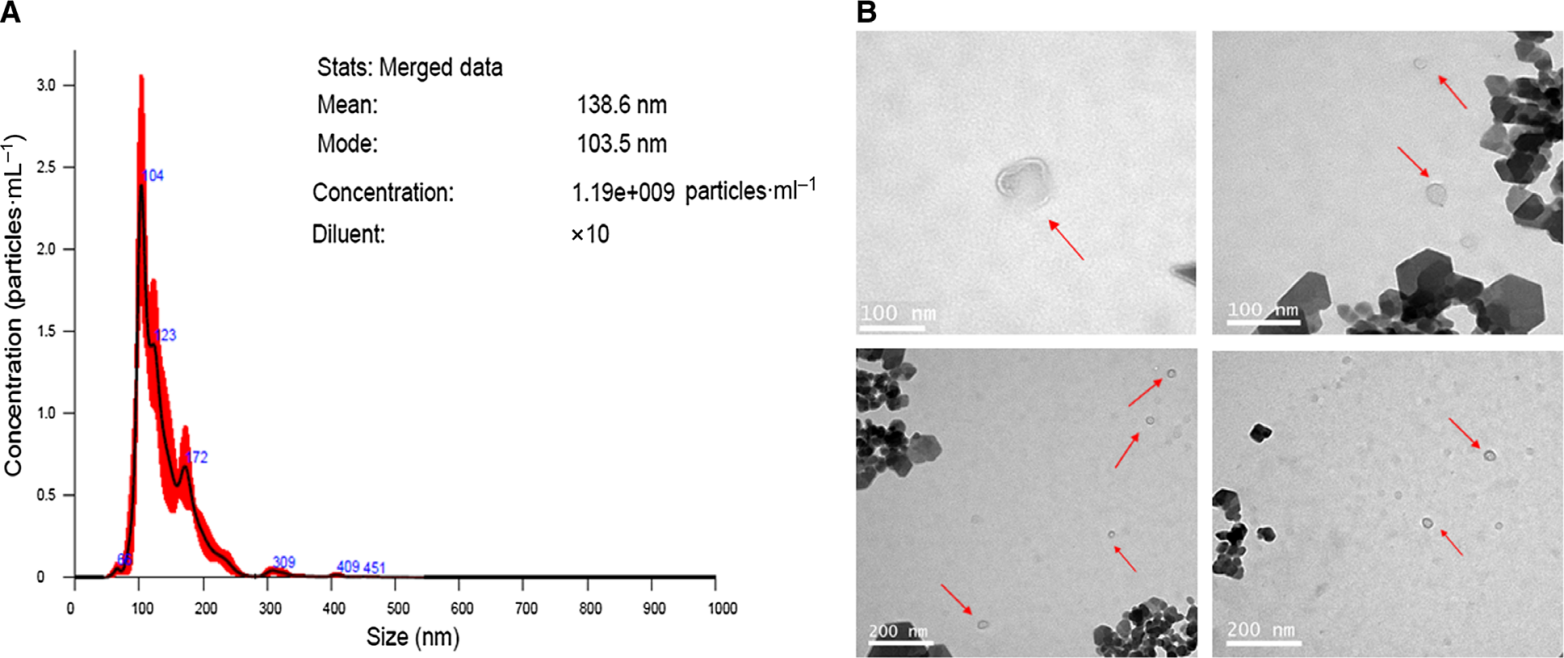
Detection of EVs isolated from human plasma. (A) Vesicle size distribution of one representative EV was measured by NTA. The mean is the average size of all vesicles measured, and the mode means the size of vesicles that occurred most frequently, that is, the size of the vesicles at the peak. (B) Representative images of EVs. Morphology of the EVs was determined by TEM. Scale bars, 100 nm (top panels); 200 nm (lower panels).

As mentioned earlier, circulating EVs were isolated from the plasma of patients with PDAC and healthy volunteers, respectively. The vesicles we obtained were examined by NanoSight NTA and TEM analyses to confirm the purification of EVs (Fig. 1). The results of NTA analysis showed that the diameter of the vesicles ranged from 30 to 140 nm, which is in line with the size of EVs (Fig. 1A). In agreement with the previously reported form of EVs, the isolated vesicles displayed a homogeneous population of clearly defined spherical vesicles under TEM (Fig. 1B), hinting that EVs were successfully purified from the plasma samples of patients with PDAC and healthy volunteers.

### Differential expression of circRNAs in EVs from the plasma of patients with PDAC

**Figure 2:**
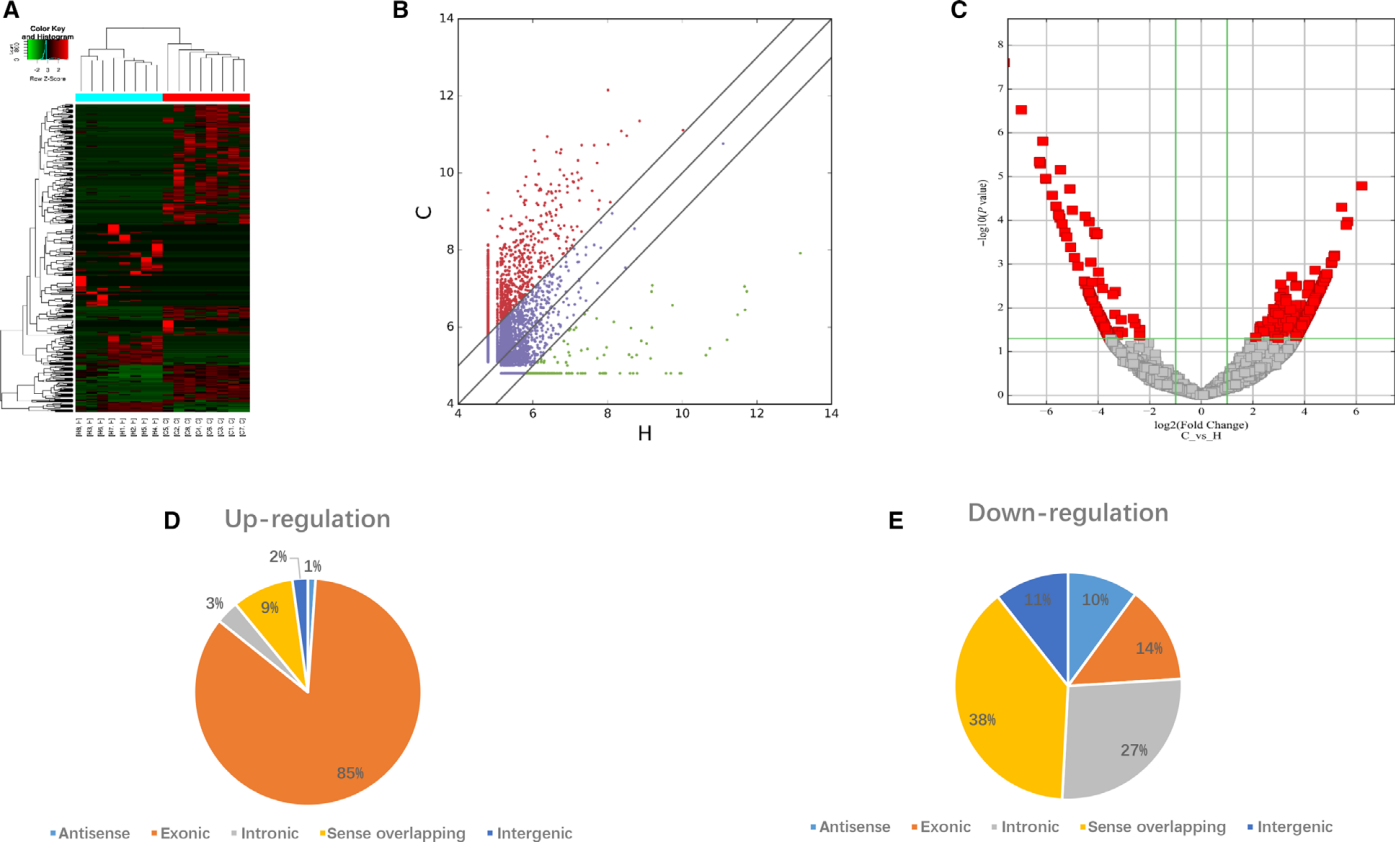
Differential expression of circRNAs in EVs between patients with PDAC and healthy volunteers. (A) Heatmap for the 453 significant differentially expressed circRNAs between EVs of PDAC and normal control. C1–C8 represent samples from patients with PDAC, and H1–H8 represent samples from healthy controls. Each row represents a circRNA, and each column represents a sample. Hierarchical clustering analysis of the samples was shown. (B) The scatterplot showed the EVs circRNA expression variation between patients with PDAC and healthy control subjects. The values of the *x* and *y* axes in the scatterplot are the averaged normalized signal values of groups of samples (log2 scaled). The oblique lines are fold change lines. The circRNAs above the top oblique line and below the bottom oblique line indicated more than 2‐fold change of circRNAs between the two groups of samples. (C) Volcano plot of the differentially expressed circRNAs. The vertical lines demark the fold change values, whereas the green horizontal line marks a *P*‐value of 0.05. The red squares in the plot represent the differentially expressed circRNAs in patients with PDAC compared with the control subjects (*P* < 0.05), corresponding to 2‐fold up and down, respectively. (D, E) Classification of the dysregulated circRNAs by a pie chart.

circRNAs in EV samples from plasma of eight patients with PDAC and eight normal healthy volunteers were sequenced, and the circRNA‐seq reads of each sample are shown in Table 2. A total of 453 significantly differentially expressed circRNAs were finally identified using the criteria of fold change ≥ 2.0 and *P* ≤ 0.05. Among these circRNAs, 274 were up‐regulated and 179 were down‐regulated. Hierarchical clustering analysis was subsequently performed to illustrate the distinguishable circRNA expression profile of the samples. As shown in Fig. 2A, the circRNAs exhibited a different expression pattern in patients with PDAC compared with the control group. According to the means of the expression values of the samples from two groups, a scatterplot was drawn to illustrate the variation of circRNA expression of the two groups (Fig. 2B). Furthermore, differentially expressed circRNAs with statistical significance (fold change > 2.0, *P* < 0.05) between the two groups were identified and filtered using a volcano plot (Fig. 2C), and the dysregulated circRNAs were summarized and classified on the basis of their categories. Among the up‐regulated circRNAs, there are 3 antisense, 232 exonic, 9 intronic, 24 sense overlapping and 6 intragenic (Fig. 2D). Among the down‐regulated circRNAs, there are 18 antisense, 25 exonic, 48 intronic, 69 sense overlapping and 19 intragenic (Fig. 2E).

**Table 2:** Top 30 differentially expressed circRNAs in pancreatic cancer. FC, fold change; FDR, false discovery rate.

circBaseID	logFC	*P*‐value	FDR	Regulation	Catalog	Chromosome	Strand	Gene name
hsa_circ_0002130	6.2167151	1.642E−5	0.0478168	Up	Exonic	chr19	−	*C3*
hsa_circ_0000896	5.6680874	0.0001058	0.1491639	Up	Exonic	chr19	−	*FARSA*
hsa_circ_0101692	5.6180826	0.0001281	0.1621948	Up	Sense overlapping	chr14	−	*EGLN3*
hsa_circ_0005882	5.4368493	4.975E−5	0.1034647	Up	Exonic	chr2	−	*STK39*
hsa_circ_0001250	5.0584905	0.0008941	0.3949192	Up	Exonic	chr22	+	*GRAMD4*
hsa_circ_0037096	4.834883	0.0018159	0.3949192	Up	Exonic	chr15	−	*PCSK6*
hsa_circ_0000128	4.8122453	0.0019492	0.3949192	Up	Exonic	chr1	+	*SCNM1*
hsa_circ_0000606	4.7943845	0.0020557	0.3949192	Up	Sense overlapping	chr15	−	*ICE2*
hsa_circ_0007177	4.7822821	0.0021337	0.3949192	Up	Exonic	chr7	+	*CCZ1*
hsa_circ_0008590	4.7757813	0.0021697	0.3949192	Up	Exonic	chr19	+	*RELB*
hsa_circ_0006404	4.7409399	0.0024077	0.3949192	Up	Exonic	chr6	+	*FOXO3*
hsa_circ_0005552	4.7304015	0.0024841	0.3949192	Up	Exonic	chr2	+	*EHBP1*
hsa_circ_0008615	4.7262769	0.0025109	0.3949192	Up	Exonic	chr19	−	*PPP1R13L*
hsa_circ_0074368	4.7229576	0.0025364	0.3949192	Up	Exonic	chr5	+	*ARHGAP26*
hsa_circ_0001821	4.7214258	0.002551	0.3949192	Up	Exonic	chr8	+	*PVT1*
hsa_circ_0103896	−4.353607	0.0045003	0.3949192	Down	Exonic	chr15	+	*G027572*
hsa_circ_0006662	−4.306561	0.0052713	0.3949192	Down	Exonic	chr2	+	*GLS*
hsa_circ_0092763	−4.280169	0.0057533	0.3949192	Down	Exonic	chr10	+	*ATRNL1*
hsa_circ_0035432	−4.232185	0.0025918	0.3949192	Down	Exonic	chr15	+	*CGNL1*
hsa_circ_0094190	−4.033311	0.0120721	0.3949192	Down	Exonic	chr10	+	*KAT6B*
hsa_circ_0013652	−3.937573	0.0157776	0.3949192	Down	Exonic	chr1	−	*CSDE1*
hsa_circ_0119741	−3.895892	0.0178304	0.3949192	Down	Exonic	chr2	+	*LCLAT1*
hsa_circ_0091122	−3.824523	0.0214853	0.3949192	Down	Exonic	chrX	+	*ATP7A*
hsa_circ_0042341	−3.738547	0.0274489	0.3949192	Down	Exonic	chr17	−	*SHMT1*
hsa_circ_0138455	−3.683197	0.0330624	0.3949192	Down	Exonic	chr9	+	*FOCAD*
hsa_circ_0130728	−3.630147	0.0379107	0.3949192	Down	Exonic	chr6	−	*MOXD1*
hsa_circ_0091682	−3.612208	0.0396748	0.3949192	Down	Exonic	chrX	+	*MTM1*
hsa_circ_0008678	−3.570223	0.0451113	0.3949192	Down	Exonic	chr9	+	*FOCAD*
hsa_circ_0116742	−3.539399	0.0475604	0.3949192	Down	Exonic	chr22	−	*EFCAB6*
hsa_circ_0019985	−3.539399	0.0475604	0.3949192	Down	Exonic	chr10	−	*SORCS1*

### Bioinformatics analysis of the differentially expressed circRNAs

**Figure 3:**
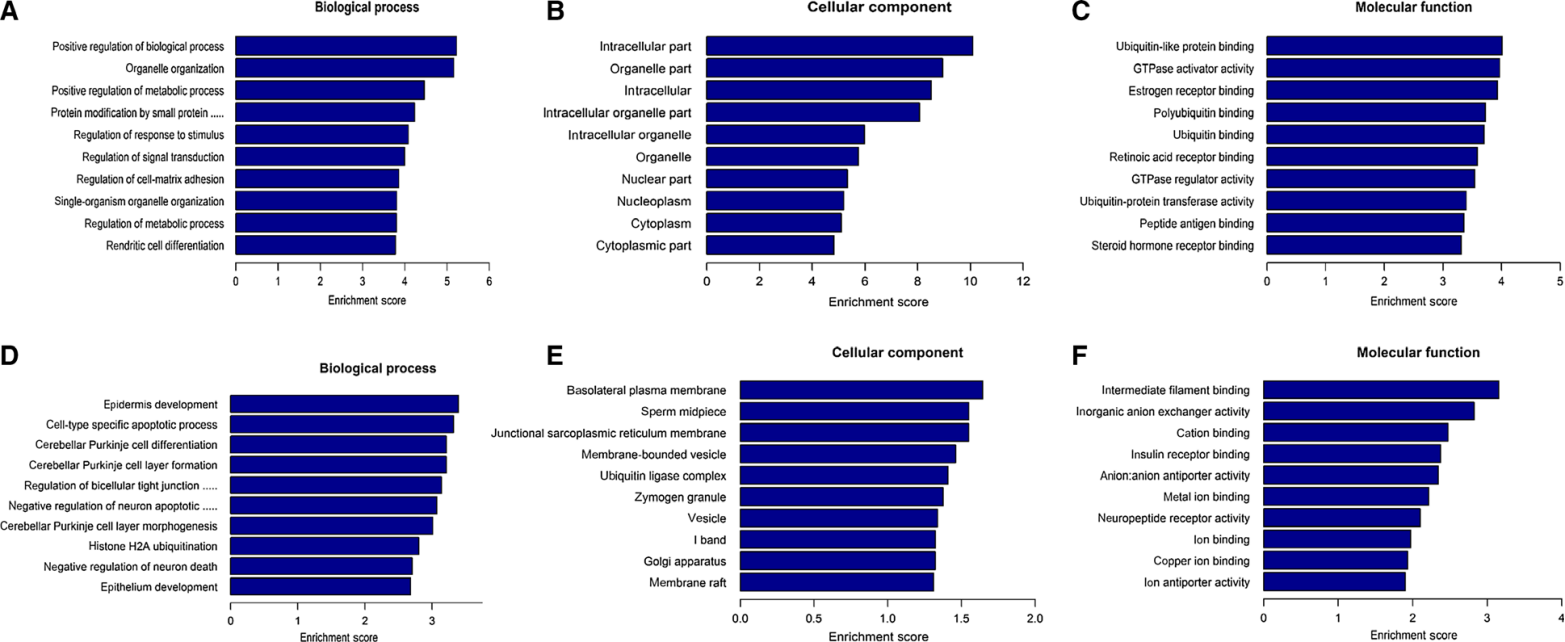
GO analysis of the dysregulated EVs circRNAs in patients with PDAC. The horizontal axis represents the enrichment scores for the GO terms, and each column represents a GO term. (A–C) GO analysis of the up‐regulated circRNAs was shown in terms of (A) BP, (B) CC and (C) molecular function, respectively. (D–F) GO analysis of the down‐regulated circRNAs was shown in terms of (D) BP, (E) CC and (F) molecular function, respectively.

circRNA‐derived genes were subjected to GO analysis to annotate and speculate the functional roles of the differentially expressed circRNAs in terms of biological processes (BPs), cellular components (CCs) and molecular functions (Fig. 3). GO analysis of BPs showed that the differentially expressed circRNAs were significantly associated with positive regulation of BP, organelle organization, positive regulation of metabolic progress, epidermis development, cell‐type‐specific apoptotic process and Purkinje cell differentiation. In regard to CC, these circRNAs exhibited a strong relationship with intracellular part, organelle part, intracellular, basolateral plasma membrane, sperm midpiece and junctional sarcoplasmic reticulum membrane. As for molecular function, they were associated with ubiquitin‐like protein binding, GTPase activator activity, estrogen receptor binding, intermediate filament binding, inorganic anion exchanger activity and cation binding.

**Figure 4:**
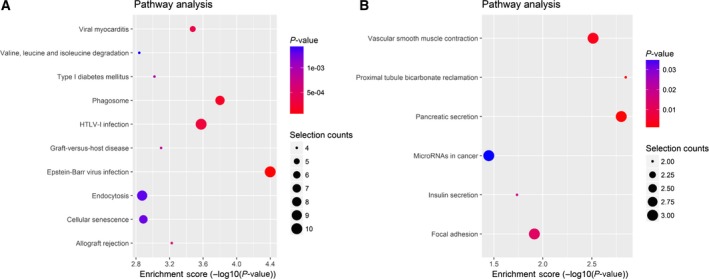
KEGG pathway analysis of the dysregulated EVs circRNAs in patients with PDAC. (A) Top ten annotated significant pathways of the up‐regulated circRNAs were shown. (B) Top six annotated significant pathways of the down‐regulated circRNAs were shown. The horizontal axis is the −log*P* (logarithm of *P*‐value) for the pathway, and the vertical axis is the pathway category. *P* < 0.05 was considered significant.

Further KEGG pathway analysis found significantly enriched pathways with the top ten enrichment scores [−log10 (*P*‐value)] (Fig. 4). Epstein‐Barr virus infection, phagosome, human T lymphotropic virus type I infection, viral myocarditis, allograft rejection, graft‐versus‐host disease, type 1 diabetes mellitus, cellular senescence, endocytosis, and valine, leucine and isoleucine degradation were included in the top ten pathways of the up‐regulated circRNAs. Of these pathways, the Epstein‐Barr virus infection had the highest enrichment score. On the other hand, the top six pathways of down‐regulated circRNAs were proximal tubule bicarbonate reclamation, pancreatic secretion, vascular smooth muscle contraction, focal adhesion, insulin secretion and miRNAs in cancer. The proximal tubule bicarbonate reclamation had the highest enrichment score among these signaling pathways.

### Validation of the accuracy of the circRNA‐seq data

**Figure 5:**
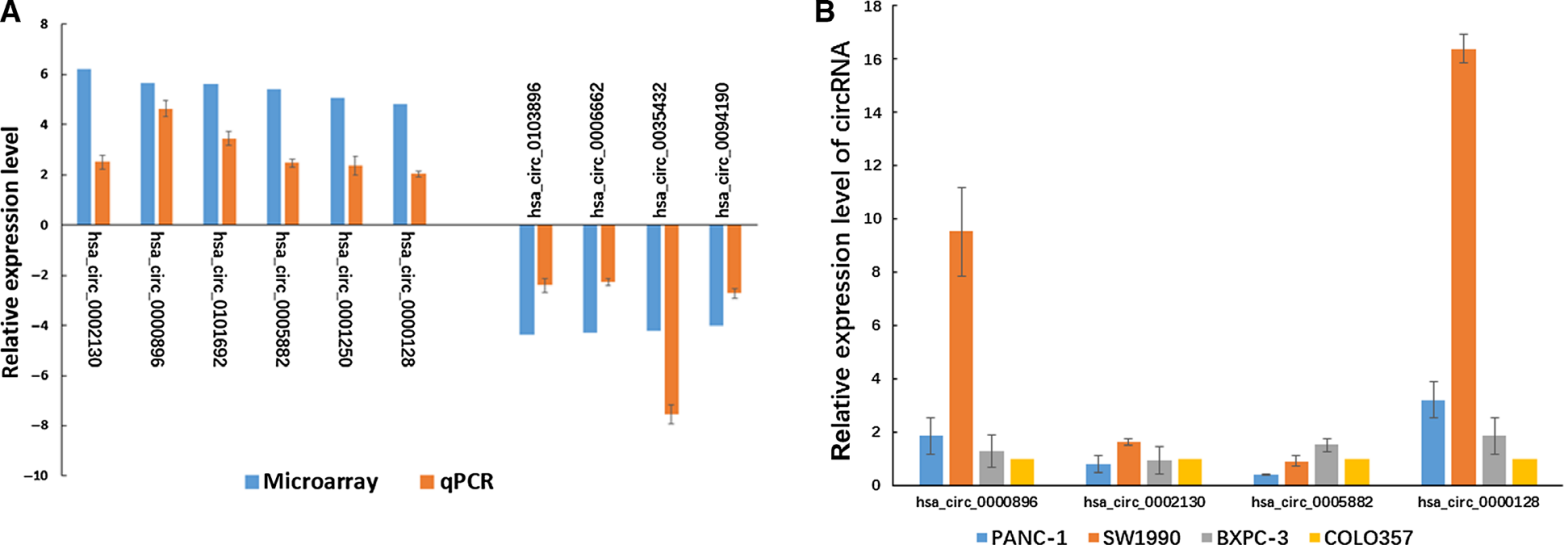
(A) Validation of the differentially expressed circRNAs obtained from circRNA‐seq data. The expression of the top eight up‐regulated and top five down‐regulated circRNAs obtained from RNA‐seq was validated by quantitative real‐time PCR analysis using EVs of 14 patients with PDAC and healthy control subjects. (B) Expression levels of four up‐regulated circRNAs were detected in four kinds of human pancreatic cancer cell lines. The *y* axis of the columns is the log2‐transformed median fold changes in expression, and data were presented as the mean and standard deviation values. These experiments were repeated three times.

According to the ranking order of the magnitude of fold changes and *P*‐values of the differentially expressed circRNAs, five down‐regulated circRNAs and eight up‐regulated circRNAs were selected (Table 2), and quantitative real‐time PCR analysis was performed to confirm the expression profiles of these circRNAs (Fig. 5). The results indicated that 10 out of 13 circRNAs (*P* < 0.05) showed the same change trends and statistical significance as the circRNA‐seq data. These findings demonstrated that the circRNA expression profiles obtained from RNA‐seq were consistent with the results of quantitative real‐time PCR analysis.

Furthermore, to demonstrate that the EVs circRNAs mentioned earlier were derived from pancreatic cancer, four up‐regulated circRNAs selected from Table 2 were validated by quantitative real‐time PCR analysis using four human PC cell lines. On the basis of the results, these selected circRNAs all expressed in PC cell lines, especially has_circ_0000896 and has_circ_0000128 remarkably expressed in SW1990 compared with other cell lines. Further research will be carried out to identify their role in the development of pancreatic cancer.

### Construction of the circRNA‐miRNA interaction network

**Figure 6:**
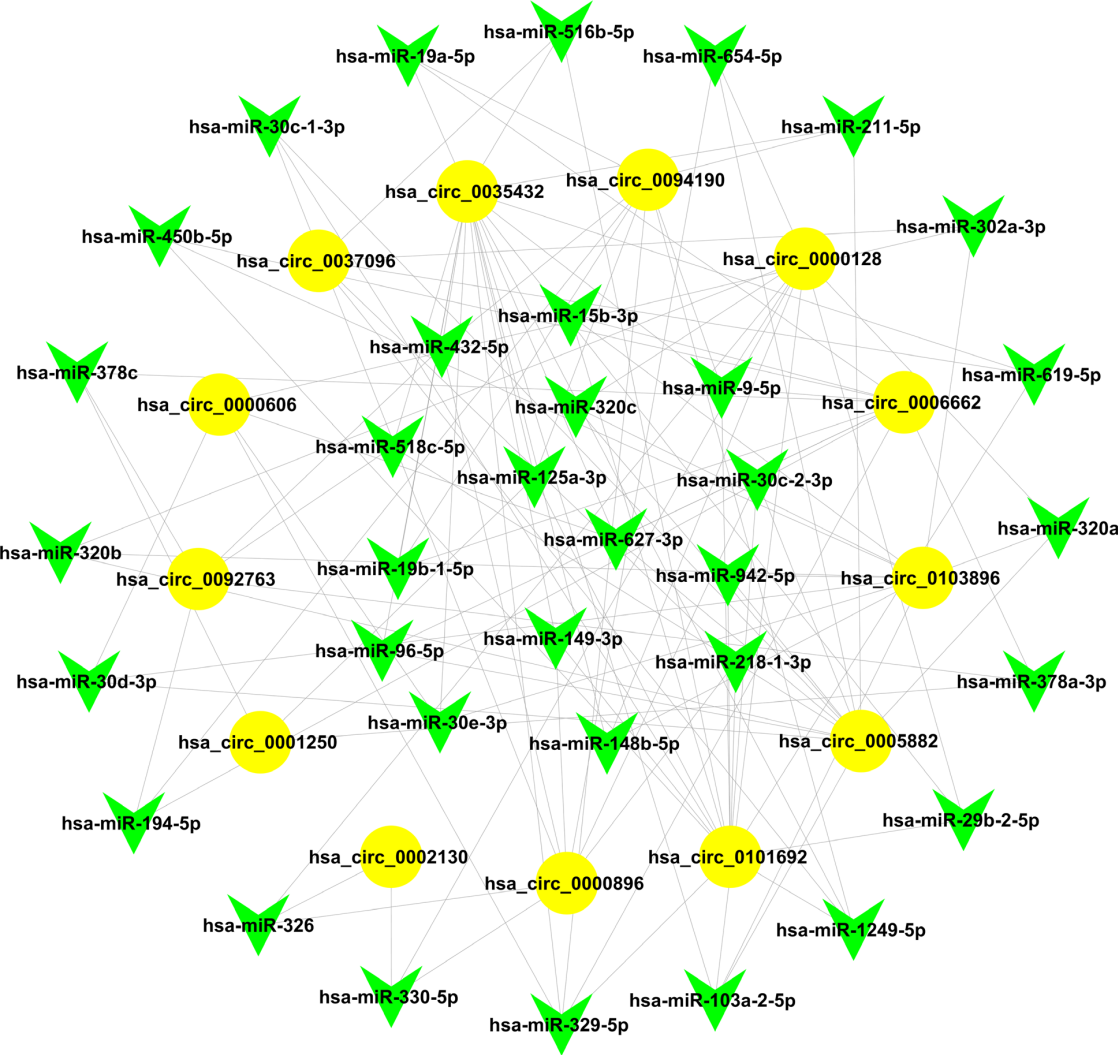
Predicted circRNA‐miRNA interaction network of the 13 selected differentially expressed circRNAs. Yellow cycles represent circRNAs, and green arrows represent miRNAs.

It has been well known that circRNAs play a key role as miRNA sponges to regulate the expression levels of other related RNAs by miRNA response elements. Therefore, we tried to identify the interaction of circRNAs and miRNAs in the next step. To theoretically predict miRNA binding sites of the circRNAs, we used miRanda and Target Scan database for conserved seed‐matching sequencing, and the 13 validated circRNAs were selected to construct the circRNA‐miRNA network using cytoscape software (Fig. 6). The network showed that hsa‐miR‐619‐5p, hsa‐miR‐6825‐5p, hsa‐miR‐3916, hsa‐miR‐6756‐5p and hsa‐miR‐4739, hsa‐miR‐6779‐5p, hsa‐miR‐6797‐5p and hsa‐miR‐6812‐5p were regulated by a greater number of circRNAs than other miRNAs.

## Discussion

Because of its important role in communicating with the tumor microenvironment, EVs have been widely reported in tumor research. In recent years, scientists have found that circulating EVs could be used as a potential biomarker for the diagnosis of PDAC 31, 32, but the molecular basis on which PDAC‐derived EVs regulate the development of PDAC requires further clarification. Herein, we delineated for the first time the expression profiles of the circRNAs derived from EVs of patients with PDAC by high‐throughput whole transcriptome sequencing and following bioinformatic analyses.

First, we isolated EVs from the plasma of eight patients with PDAC and eight healthy volunteers using the ultracentrifugation method. The collected EVs were subsequently detected by NTA and TEM analyses, and the results confirmed the size and form of these EVs.

In the next step, circRNAs from the isolated EVs were sequenced, and we found that 453 circRNAs were significantly differentially expressed in patients with PDAC compared with the healthy control subjects, including 274 up‐regulated and 179 down‐regulated. To verify the circRNA‐seq data, we selected the top 13 differentially expressed cirRNAs on the basis of the ranking order in Table 2 to conduct quantitative real‐time PCR analysis, and the results confirmed the accuracy of the circRNA‐seq data. To demonstrate that these circRNAs were derived from pancreatic cancer, the expressions of four up‐regulated circRNAs in PC cell lines were also validated by quantitative real‐time PCR analysis. Given that the release of EVs is an important mechanism for tumor cells to communicate with the tumor microenvironment, these differentially circRNAs may participate in the development of PDAC as key content of EVs.

Further GO analysis was performed to explore the potential function of these circRNAs. The data of GO analysis indicated that these circRNAs are strongly associated with a series of biological activities and metabolic processes in terms of BPs. In regard to CCs, they exhibited a strong relationship with intracellular part, organelle part and so on. As for MPs, they showed capacities to bind with specific proteins, GTPase activator activity and so on. Therefore, the functional roles of these differentially expressed circRNAs are possibly associated with normal cell activities and signal transduction.

In addition, results of the KEGG pathway analysis indicated that differentially expressed circRNAs mainly participate in several biological pathways, such as Epstein‐Barr virus infection and miRNAs in cancer. Of these pathways, the most relevant pathway is Epstein‐Barr virus infection, which is the first herpesvirus identified to be associated with a remarkably diverse range of cancer types, such as lymphoma, nasopharyngeal cancer, gastric cancer and breast cancer 33, 34, 35, 36, 37. However, its relationship with PDAC remains unclear yet. miRNAs in cancer have been a hot area of cancer research for several years, and many miRNAs are found dysregulated in different cancer types. By targeting mRNAs, miRNAs could influence a lot of cancer‐related processes such as cell growth, migration, apoptosis and metabolism 38. Because circRNAs can function as miRNA sponges to modulate miRNA activity, and considering that EVs are key players in intercellular communication between tumor cells and their microenvironment, we suspected that the differentially expressed circRNAs we identified might affect PDAC progression and cell transformation by targeting miRNAs after release from EVs. On the basis of KEGG pathway analysis, we hypothesized that these circRNAs possibly regulated PDAC progression through one or some of these pathways. However, all of our hypotheses need further verification.

In addition, the interaction between the 13 selected circRNAs and their miRNA targets were predicted by conserved seed‐matching sequencing using TargetScan and miRanda database, and a network was constructed by cytoscape software. The circRNA‐miRNA network indicated that all of the differentially expressed circRNAs have their respective miR response elements, and these data will provide direction for further studies.

In summary, the present work, for the first time, identified a series of differentially expressed circRNAs derived from EVs of patients with PDAC compared with healthy controls. GO and KEGG pathway analyses indicated potential functional roles of these circRNAs in humans, and a circRNA‐miRNA interaction network was graphed according to our predictions. Our data link EVs, circRNAs and miRNAs in PDAC together, which will provide novel targets for further studies about mechanisms of PDAC progression and therapeutic strategies against PDAC.

## Conflict of interest

The authors declare no conflict of interest.

## Author contributions

HJ and YDL planned experiments. QL, SG, HY and YL performed experiments. SZ and LP analyzed data. QL, JG and XN wrote the manuscript. All authors edited and approved the final version of the manuscript.
